# Distinct Effector Memory CD4^+^ T Cell Signatures in Latent *Mycobacterium tuberculosis* Infection, BCG Vaccination and Clinically Resolved Tuberculosis

**DOI:** 10.1371/journal.pone.0036046

**Published:** 2012-04-24

**Authors:** Toidi Adekambi, Chris C. Ibegbu, Ameeta S. Kalokhe, Tianwei Yu, Susan M. Ray, Jyothi Rengarajan

**Affiliations:** 1 Emory Vaccine Center, Emory University School of Medicine, Atlanta, Georgia, United States of America; 2 Division of Infectious Disease, Department of Medicine, Emory University, Atlanta, Georgia, United States of America; 3 Department of Biostatistics and Bioinformatics, Rollins School of Public Health, Atlanta, Georgia, United States of America; National Institute for Infectious Diseases (L. Spallanzani), Italy

## Abstract

Two billion people worldwide are estimated to be latently infected with *Mycobacterium tuberculosis* (Mtb) and are at risk for developing active tuberculosis since Mtb can reactivate to cause TB disease in immune-compromised hosts. Individuals with latent Mtb infection (LTBI) and BCG-vaccinated individuals who are uninfected with Mtb, harbor antigen-specific memory CD4^+^ T cells. However, the differences between long-lived memory CD4^+^ T cells induced by latent Mtb infection (LTBI) versus BCG vaccination are unclear. In this study, we characterized the immune phenotype and functionality of antigen-specific memory CD4^+^ T cells in healthy BCG-vaccinated individuals who were either infected (LTBI) or uninfected (BCG) with Mtb. Individuals were classified into LTBI and BCG groups based on IFN-γ ELISPOT using cell wall antigens and ESAT-6/CFP-10 peptides. We show that LTBI individuals harbored high frequencies of late-stage differentiated (CD45RA^−^CD27^−^) antigen-specific effector memory CD4^+^ T cells that expressed PD-1. In contrast, BCG individuals had primarily early-stage (CD45RA^−^CD27^+^) cells with low PD-1 expression. CD27^+^ and CD27^−^ as well as PD-1^+^ and PD-1^−^ antigen-specific subsets were polyfunctional, suggesting that loss of CD27 expression and up-regulation of PD-1 did not compromise their capacity to produce IFN-γ, TNF-α and IL-2. PD-1 was preferentially expressed on CD27^−^ antigen-specific CD4^+^ T cells, indicating that PD-1 is associated with the stage of differentiation. Using statistical models, we determined that CD27 and PD-1 predicted LTBI versus BCG status in healthy individuals and distinguished LTBI individuals from those who had clinically resolved Mtb infection after anti-tuberculosis treatment. This study shows that CD4^+^ memory responses induced by latent Mtb infection, BCG vaccination and clinically resolved Mtb infection are immunologically distinct. Our data suggest that differentiation into CD27^−^PD-1^+^ subsets in LTBI is driven by Mtb antigenic stimulation *in vivo* and that CD27 and PD-1 have the potential to improve our ability to evaluate true LTBI status.

## Introduction

Tuberculosis (TB) remains a serious public health threat and was responsible for about 1.4 million deaths in 2010 [1]. Remarkably, it has been estimated that 2 billion people worldwide are latently infected with *Mycobacterium tuberculosis* (Mtb), with no symptoms of disease [2]. While healthy individuals with latent Mtb infection (LTBI) successfully control infection, Mtb can persist within individuals for decades before reactivating to cause active TB disease when the host is immune-compromised [3], [4], [5], [6]. The failure to fully clear bacteria in LTBI [6], [7], therefore represents a vast reservoir for increased transmission of TB, particularly in populations co-infected with Human Immunodeficiency Virus (HIV) [2], [8], [9]. Vaccination with *Mycobacterium bovis* Bacille Calmette-Guerin (BCG) confers consistent and reliable protection against miliary TB and TB meningitis in infants [10], [11]. However, BCG has variable - mostly poor- efficacy in protecting against pulmonary TB disease in adults and children [12] and does not prevent LTBI. In the United States, detection of LTBI in healthy foreign-born persons is an important public health challenge as a large proportion of foreign-born U.S. residents have a history of BCG vaccination. It is estimated that 10–15 million individuals have LTBI and that 63% of these individuals are foreign-born [13], [14]. Notably, BCG-vaccinated individuals who are uninfected with Mtb as well as those who have LTBI can show positive reactivity to the Tuberculin Skin Test (TST), which is widely used for LTBI diagnosis. The TST is not specific for Mtb infection since it measures memory responses to intradermal injection of Mtb purified protein derivative (PPD), which cross-reacts with Mtb and BCG strains. While both latent Mtb infection and BCG vaccination can induce mycobacteria-specific memory CD4^+^ T cells in healthy individuals, the similarities and differences between the long-lived memory CD4^+^ T cell compartments induced by LTBI versus BCG vaccination are unclear. Although Mtb-specific interferon gamma release assays (IGRAs) like QuantiFERON and T-SPOT.TB discriminate between LTBI and BCG vaccination, they do not provide information about the phenotype and quality of memory responses induced by latent Mtb infection or BCG vaccination. We reasoned that detailed understanding of how LTBI and BCG vaccination shape long-lived memory CD4^+^ T cell compartments in healthy individuals should provide important insights into the nature of infection- and vaccine-induced immunity.

Several studies have established that the production of Th_1_ cytokines like IFN-γ and TNF-α by antigen-specific CD4^+^ T cells is critical for controlling Mtb infection and containing bacteria within lung granulomas [15], [16]. Indeed, lowering of CD4^+^ counts is known to significantly increase the risk of TB disease [17]. CD4^+^ T cells that survive the acute phase of infection are retained as memory cells and Mtb-specific memory CD4^+^ T cells are central to prevent progression to TB in healthy latently infected individuals [15], [16]. Polyfunctional CD4^+^ T cells that simultaneously express the three cytokines IFN-γ, TNF-α and IL-2 are thought to be associated with the control of intracellular pathogens [18] including Mtb [19]. Furthermore, many studies have revealed that the differentiation and function of memory T cell subsets in healthy individuals is directly shaped by antigenic stimulation induced by vaccination or latent viral infection [20], [21], [22]. However, we lack detailed insights into how the immune phenotype and quality of long-lived memory CD4^+^ T cell compartments in healthy individuals is shaped and influenced by latent Mtb infection versus BCG vaccination.

In this study, we performed detailed characterization of the immune phenotype and functionality of antigen-specific memory CD4^+^ T cells in healthy BCG-vaccinated individuals who were either infected (LTBI) or uninfected with Mtb (BCG). We show that CD27 and PD-1 expression on antigen-specific memory CD4^+^ T cells could predict LTBI versus BCG status in healthy individuals. Interestingly, we found that CD27 and PD-1 also distinguish LTBI individuals from those who have clinically resolved Mtb infection after anti-tuberculosis treatment. These studies show that the CD4^+^ memory responses induced by latent Mtb infection, BCG vaccination and clinically resolved Mtb infection are immunologically distinct and suggest that differentiation into CD27^−^ PD-1^+^ subsets is driven by persistent antigenic stimulation by Mtb *in vivo*. CD27 and PD-1 have the potential to improve our ability to evaluate Mtb infection status in healthy individuals.

## Results

### IFN-γ Elispot to identify individuals with memory responses to Mtb infection

**Figure 1:**
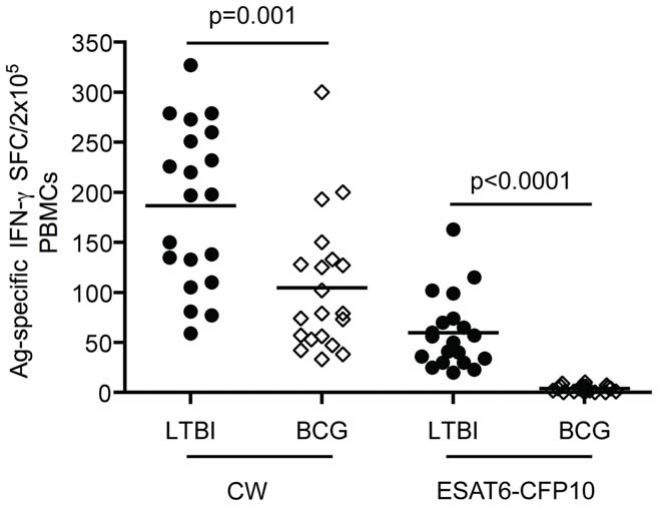
Identification of LTBI individuals by IFN-γ ELISPOT assays. PBMC isolated from 40 healthy BCG-vaccinated individuals were stimulated with Mtb cell wall antigens (CW) or peptide pools derived from Mtb-specific proteins ESAT-6 and CFP-10 (10 mg/ml). Data are represented as spot forming cells (SFC) per 2×10^5^ PBMC. Each data point corresponds to a single donor.

Using IFN-γ ELISPOT assays, we screened PBMCs from 80 healthy adults for reactivity to Mtb CW antigens, which are a mixture of Mtb cell wall components [23], and to peptide pools derived from the Mtb-specific proteins ESAT-6 and CFP-10. All enrolled individuals were born outside the United States and had BCG vaccination histories. CW antigens cross-react with the *M. bovis* BCG strain and therefore CW-specific memory responses will be present in individuals who were latently infected with Mtb (LTBI) as well as in BCG-vaccinated individuals (BCG) that are not infected with Mtb. Therefore, only individuals with Mtb-specific memory T cells will respond to stimulation with ESAT-6 and CFP-10 peptide. **Figure 1** shows that individuals were classified as LTBI (n = 20) when they showed Mtb-specific memory T cell responses, i.e. they had positive responses to both CW and ESAT-6/CFP-10 peptides. Those that showed reactivity to CW antigens but not to ESAT-6/CFP-10 peptides were classified as BCG (n = 20). While both LTBI and BCG groups exhibited strong IFN-γ responses to CW antigens, individuals in the LTBI group had higher frequencies of antigen-specific cells as reflected by the percentages of spot forming cells (SFC) in the Elispot assay (**Fig. 1**). Among BCG-vaccinated individuals tested, 40 were not further studied as they showed no reactivity to any of the mycobacterial antigens tested, consistent with the documented waning of immune memory in BCG-vaccinated individuals over time [24]. Five non-BCG-vaccinated healthy individuals who did not show reactivity to either CW or ESAT-6/CFP-10 peptides served as negative controls (**Fig. S1**).

### Phenotypic and functional characterization of CD4^+^ T cells in LTBI and BCG individuals

**Figure 2:**
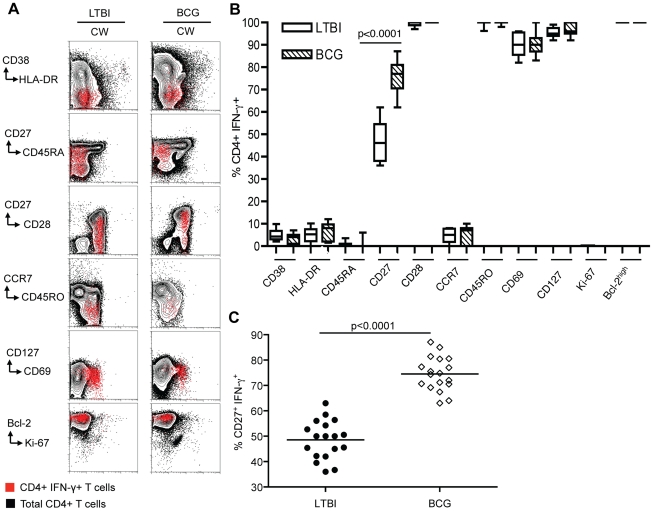
Characterization of *Mtb* antigen-specific memory CD4^+^ T cells in LTBI and BCG individuals using multiparameter flow cytometry. (A) Immunophenotyping of memory CD4^+^ T cells from LTBI and BCG individuals. Data from two representative donors are shown. The indicated cell surface markers on CW-stimulated PBMC gated on total CD4^+^ T cells were assessed by flow cytometry (black) and on IFN-γ producing cells were assayed by intracellular staining (red). (B) Graphical summary of immunophenotypic analyses. The expression of each marker is expressed as a percentage of the total antigen-specific IFN-γ^+^ CD4^+^ T cells in LTBI (n = 18) and BCG (n = 18) groups (C). Expression of CD27 on antigen-specific memory CD4^+^ T cells from LTBI (n = 18) and BCG (n = 18). The percentage of antigen-specific CD4^+^ T cells is significantly different in LTBI and BCG groups (p<0.0001).

We used multi-parameter flow cytometry for detailed immunophenotypic analyses to evaluate the quality of long-lived Mtb-specific memory CD4^+^ T cells in PBMC from the LTBI and BCG groups. We assessed their homeostatic turnover, differentiation state and cytokine production upon stimulation with Mtb CW antigen. Representative data are shown in **Fig. 2A** and summarized in **Fig. 2B**. Memory CD4^+^ T cells in both groups were in a resting state as they did not express CD38 and HLA-DR, which are markers of immune activation [20], [25], and were in a non-cycling state as they were negative for Ki-67, which is associated with cycling T cells [26]. The expression of the anti-apoptotic protein Bcl-2 suggests that these cells were not destined to undergo apoptosis [27]. Both LTBI and BCG groups harbored effector memory subsets as indicated by expression of CD127 (IL-7Rα), which is selectively re-expressed on memory T cells [28], but no co-expression of CCR7, a chemokine receptor that is expressed preferentially on central memory subsets [29]. Examination of the differentiation state of the antigen-specific effector memory CD4^+^ T cells in LTBI and BCG groups showed that both exhibited a CD45RA^−^ CD28^+^ phenotype (**Fig. 2A, B**). However, expression of the costimulatory marker CD27 was heterogeneous between LTBI and BCG groups (**Fig. 2A–C**). Since loss of CD27 expression denotes late-stage differentiation during the step-wise antigen-driven maturation of human effector memory T cells [30], [31], [32], our data suggest that the presence of antigen in LTBI drives maturation of effector memory CD4^+^ T cells to later stages of differentiation than in the BCG group.

**Figure 3:**
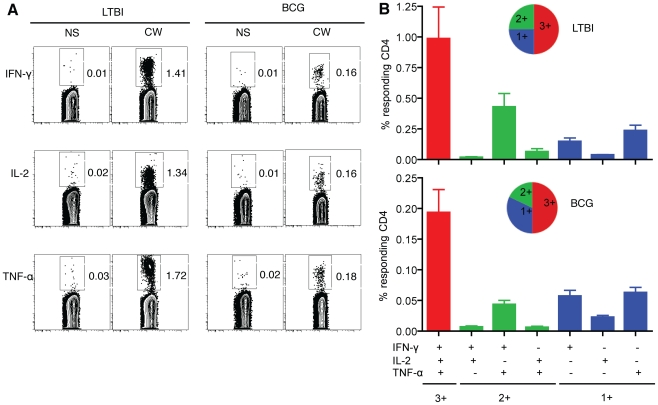
Cytokines production by effector memory CD4^+^ T cells from LTBI and BCG individuals. (A) Functional profile of IFN-γ, IL-2 and TNF-α CD4^+^ T cells are shown in representative LTBI and BCG individuals. (B) Polyfunctional cytokine production by memory CD4^+^ T cells from LTBI and BCG. Data are represented as the mean percentage of responding CD4^+^ T cells that are triple producers (3+), double producers (2+) or single producers (1+) of IFN-γ, TNF-α, and IL-2 and summarized by the pie charts. Each slice of the pie represents the fractions of the total response that consists of CD4^+^ T cells positive for a given function.

We next assessed the ability of CW-specific memory CD4^+^ T cells from LTBI and BCG groups to simultaneously express IFN-γ, TNF-α and IL-2 (**Fig. 3A**). This analysis categorized cytokine-positive cells into seven different subsets consisting of triple (3+), double (2+) and single (1+) cytokine-producing populations (**Fig. 3B**). The expression patterns of 3+, 2+ and 1+ were similar between LTBI and BCG although the BCG group had lower frequencies of antigen-specific cytokine-producing cells (**Fig. 3A, B**). Polyfunctional cytokine-producing cells that simultaneously produced IFN-γ, TNF-α and IL-2 dominated the cytokine response in both LTBI and BCG individuals. Cells expressing IFN-γ/TNF-α dominated the 2+ populations (∼90%) and ∼60% of the 1+ populations corresponded to TNF-α producers.

### CD27^+^ and CD27^−^ antigen-specific effector memory CD4^+^ T cells exhibit polyfunctional cytokine responses in LTBI and BCG individuals

**Figure 4:**
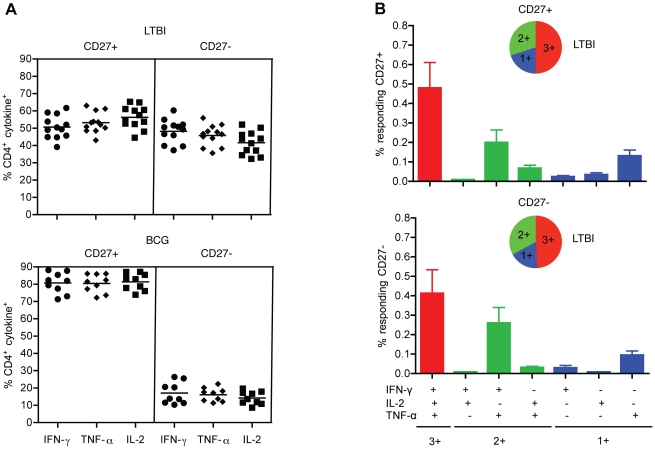
CD27^+^ and CD27^−^ antigen-specific memory CD4^+^ T cell subsets are polyfunctional. (A) Distribution of CD27^+^ and CD27^−^ memory CD4^+^ T cells from LTBI (n = 12) and BCG (n = 9) that produce IFN-γ, TNF-α or IL-2 upon stimulation with CW antigen. The antigen-specific T cells are CD28^+^, CCR7^−^ and CD45RA^−^. Each data point corresponds to a single donor. (B) Polyfunctional cytokine responses from CD27^+^ and CD27^−^ subsets in LTBI. Data are represented as the mean percentage of responding CD4^+^ T cells that are triple producers (3+), double producers (2+) or single producers (1+) of IFN-γ, TNF-α, and IL-2 and summarized by the pie charts. Each slice of the pie represents the fractions of the total response that consists of CD4^+^ T cells positive for a given function.

To assess whether CD27 expression influences the functional capacity of antigen-specific memory CD4^+^ T cells, we compared the functional capacities of CD27^+^ and CD27^−^ subsets in BCG and LTBI individuals. Both CD27^+^ and CD27^−^ subsets were capable of producing IFN-γ, TNF-α and IL-2 in response to antigenic stimulation. **Figure 4A** shows that LTBI individuals had higher frequencies of CD27^−^ late-stage differentiated antigen-specific memory CD4^+^ T cells (∼50%), compared to the BCG group which had only ∼20% (p<0.0001). Moreover, both CD27^+^ and CD27^−^ subsets in LTBI displayed similar polyfunctional cytokine profiles (**Fig. 4B**). The expression patterns of 3+ account for 49–51%, 2+ for 18–20% and 1+ for 30–33% of the total cytokine response. Cells expressing IFN-γ/TNF-α were dominant (∼80%) among the 2+ populations and cells expressing only TNF-α were dominant (∼60%) among the 1+ populations (**Fig. 4B**). While the loss of CD27 expression in LTBI suggests that the presence of antigen drives maturation of effector memory CD4^+^ T cells to later stages of differentiation than in the BCG group, our data indicate that this loss does not compromise the functional capacity of these CD4^+^ T cells.

### High frequencies of PD-1-expressing Mtb-specific CD4^+^ T cells in LTBI

**Figure 5:**
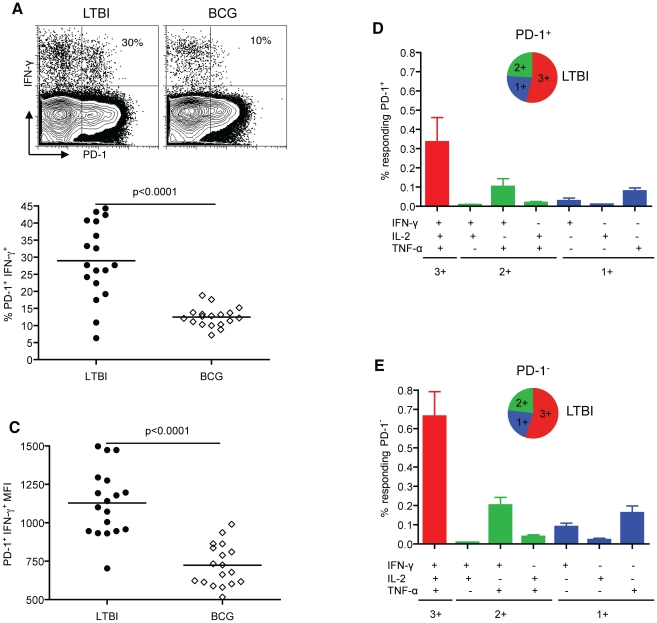
Higher frequencies of PD-1 expression on antigen-specific memory CD4^+^ T cells in LTBI relative to BCG individuals. (A) A representative plot of CD4^+^ T cells expressing PD-1 upon CW stimulation, displaying the percentage of antigen-specific IFN-γ producing cells. The summary of percentage (B) and MFI (C) of PD-1 expression on antigen-specific memory CD4^+^ T cells in LTBI (n = 18) and BCG (n = 18) groups. Each data point corresponds to a single donor. Polyfunctional cytokine production by memory PD-1^+^ (D) and PD-1^−^ (E) T cells from LTBI. Data are represented as the mean percentage of responding PD-1^+^ or PD-1^−^ T cells that are triple producers (3+), double producers (2+) or single producers (1+) of IFN-γ, TNF-α, and IL-2 and summarized by the pie charts. Each slice of the pie represents the fractions of the total response that consists of PD-1^+^ or PD-1^−^ cells positive for a given function.

To further investigate whether the late-stage differentiation of the antigen-specific CD4^+^ T cells in LTBI is shaped by the presence of Mtb, we examined the expression of PD-1 [33], [34], [35]. We assessed PD-1 expression on antigen-specific CD4^+^ T cells from LTBI and BCG individuals and found that the frequencies of PD-1 expression on IFN-γ-producing CD4^+^ T cells were significantly higher in LTBI than BCG individuals. The mean frequency of PD-1^+^ CD4^+^ T cells in LTBI was 2.3-fold greater than in the BCG group (mean±SEM 29.0±2.6% *vs* 12.5±0.7%, p<0.0001) (**Fig. 5A, B**). Similarly, the PD-1 mean fluorescence intensity (MFI) on antigen-specific IFN-γ^+^ CD4^+^ T cells was significantly higher in LTBI compared to BCG (mean±SEM 1129±52 *vs* 728±33; p<0.0001) (**Fig. 5C**). Overall, the antigen-specific CD4^+^ T cells show intermediate levels of PD-1 expression. There was no significant difference in PD-1 expression on bulk CD4^+^ T cells from LTBI and BCG groups (**Fig. S2**). The PD-1 expression shown in **Figure 5A and 5B** is not a result of up-regulation in cell culture as PD-1 was not induced *ex vivo* in LTBI or BCG groups until 48 hours of stimulation (data not shown). Since PD-1 expression has been shown to correlate with antigen presence, these data suggest that the higher frequencies of PD-1-expessing memory CD4^+^ T cells in LTBI compared to the BCG group reflect ongoing antigenic stimulation of memory CD4^+^ T cells in LTBI.

To examine whether PD-1 expression influences the capacity of antigen-specific cells to produce these cytokines, we analyzed the cytokine profile of PD-1^+^ and PD-1^−^ effector memory populations. We found that both PD-1^+^ and PD-1^−^ cells produce IFN-γ, TNF-α and IL-2 in response to CW stimulation and are polyfunctional (**Fig. 5D, E**). The 3+ profile was predominant in both groups (∼50%) followed by 2+ (∼25%) and 1+ (∼25%). The frequency of cells with the 3+ cytokine profile was ∼2-fold lower in PD-1^+^ compared to PD-1^−^ (p = 0.01) (**Fig. 5D, E**). These data demonstrate that intermediate levels of PD-1 expression in healthy LTBI individuals do not impact the capacity of CD4^+^ T cells to produce IFN-γ, TNF-α and IL-2 upon stimulation.

### PD-1 expression correlates with loss of CD27 expression on antigen-specific CD4^+^ T cells

**Figure 6:**
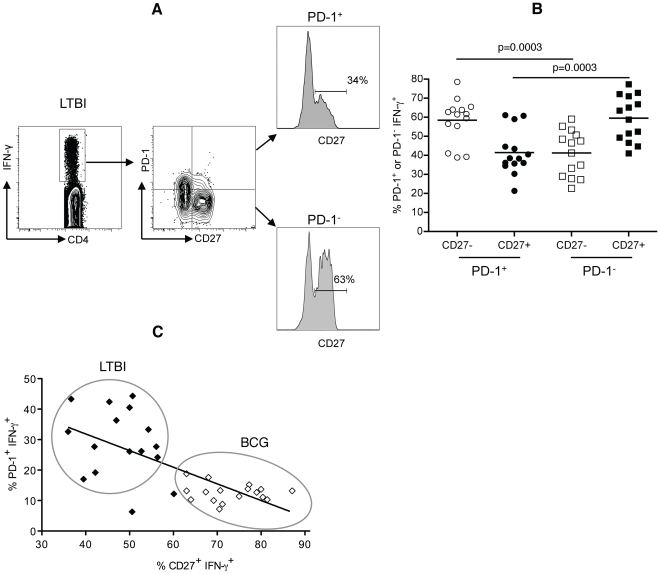
PD-1 expression correlates with loss of CD27 expression on antigen-specific memory CD4^+^ T cells. (A) Gating strategy and representative histograms showing CD27 expression on PD-1^+^ and PD-1^−^ antigen-specific IFN-γ^+^ CD4^+^ T cells in PBMC from one LTBI individual stimulated with CW antigens. (B) Frequencies of CD27^+^ and CD27^−^ subsets on PD-1^+^ and PD-1^−^ antigen-specific IFN-γ^+^ CD4^+^ T cells in LTBI individuals (n = 16) (C) PD-1 expression is inversely correlated with CD27 expression on antigen-specific CD4^+^ T cells from LTBI (n = 16) and BCG (n = 17) individuals (r = −0.69, p<0.0001). Circles highlight the data points corresponding to LTBI and BCG individuals.

We have shown that in LTBI, ∼50% of the CCR7^−^CD45RA^−^ subsets have lost CD27 expression and are therefore in late stages of antigen-driven memory CD4^+^ T cell differentiation. Moreover, a significant proportion of the memory CD4^+^ T cells express PD-1, which is typically induced by the antigen presence. We next investigated the relationship between PD-1 and CD27 on antigen-specific IFN-γ^+^ memory CD4^+^ T cells in LTBI. We examined the level of CD27 expression on PD-1^+^ and PD-1^−^ antigen-specific IFN-γ^+^ CD4^+^ T cells in LTBI individuals (**Fig. 6A**). We observed that CD27^+^ subsets were higher in PD-1^−^ T cells compared to PD-1^+^ (mean±SEM 59.5%±3.2 vs 41.4%±3.1, p<0.0003) (**Fig. 6B**), indicating that in LTBI, PD-1^+^ T cells are more differentiated than PD-1^−^ antigen-specific memory CD4^+^ T cells. Consistent with these observations, we found a significant inverse correlation between the expression of PD-1 and the expression of CD27 on IFN-γ-producing CD4^+^ T cells across LTBI and BCG groups (r<−0.69, p<0.0001) (**Fig. 6C**). Late-stage differentiated CD27^−^ PD-1^+^IFN-γ^+^ cells were more likely to be associated with LTBI individuals while the early/intermediate-stage differentiated CD27^+^ PD-1^−^IFN-γ^+^ cells were more likely to be associated with BCG individuals (**Fig. 6C**). These data suggest that PD-1 expression correlates with the stage of differentiation.

### CD27 and PD-1 expression predict LTBI versus BCG status and distinguish latent infection from clinically resolved Mtb infection

**Figure 7:**
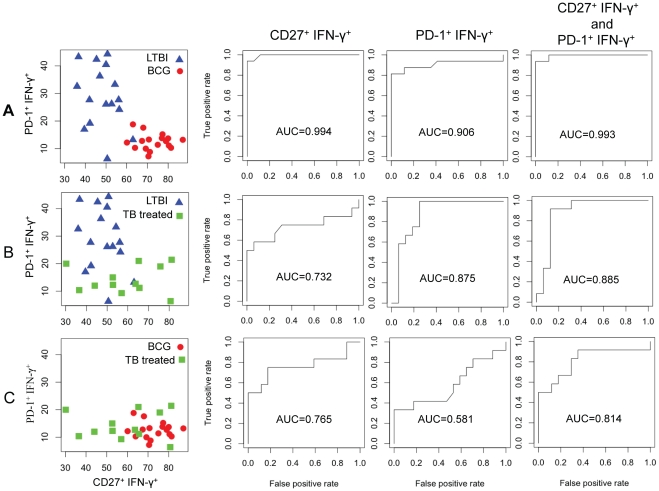
Statistical modeling to evaluate the predictive value of CD27 and PD-1 in healthy individuals. The frequencies of PD-1^+^ IFN-γ^+^ and CD27^+^ IFN-γ^+^ T cells for the following pairs (A) LTBI versus BCG, (B) LTBI versus TB-treated and (C) BCG versus TB-treated, are graphically represented. Receiver Operating Characteristic (ROC) curve analysis and logistic regression models were used to evaluate the predictive value of CD27 and PD-1 individually and in combination. The area under the curve (AUC) indicates the predictive value for each marker.

Our data show that LTBI is characterized by high frequencies of PD-1^+^CD27^−^ antigen-specific effector memory subsets while BCG-vaccinated individuals have predominantly PD-1^−^ CD27^+^ antigen-specific subsets (**Fig. 7A**). We used statistical models to evaluate the utility of CD27 and PD-1 in predicting LTBI versus BCG status in healthy individuals. To evaluate the predictive value of CD27 and PD-1 individually, we used Receiver Operating Characteristic (ROC) curve analysis and logistic regression for both markers in combination. We found that either CD27 or PD-1 or both in combination correctly identify LTBI and BCG groups with high predictive values (AUC = 0.994, 0.906 and 0.993; respectively), indicating that either CD27 or PD-1, or both markers, can discriminate between the two groups (**Fig. 7A**).

The diagnosis of LTBI is based on information gathered from the medical history of an individual, TST and/or IGRA results, chest radiograph, physical examination and in certain circumstances, sputum examinations to rule out active TB. However, individuals who have successfully completed anti-tuberculosis treatment remain TST and/or IGRA positive and cannot be distinguished from LTBI by diagnostic tests alone. We next investigated the ability of CD27 and PD-1 to discriminate between LTBI individuals and healthy individuals who were previously treated for TB and have clinically resolved Mtb infection. We also included the BCG individuals in this investigation. Healthy TB treated individuals who were asymptomatic and sputum-negative were enrolled ≥2 years after completing treatment for active TB. **Figure 7B** shows a graphical representation of the frequencies of PD-1^+^ and CD27^+^ antigen-specific memory CD4^+^ T cells in LTBI versus TB treated individuals. Using ROC analysis, we found that PD-1 alone effectively distinguished TB-treated individuals from LTBI (AUC = 0.875), suggesting that PD-1 may have utility as a marker of Mtb presence (**Fig. 7B**). CD27 was slightly less effective when tested singly (AUC = 0.732). However the predictive value of PD-1 when tested in combination with CD27 improved (AUC = 0.885) (**Fig. 7B**). In contrast, PD-1 was less informative in distinguishing between BCG and TB-treated individuals (AUC = 0.581) (**Fig. 7C**). This was consistent with the low frequencies of PD-1^+^ antigen-specific T cells in BCG and TB treated groups (**Fig. 7C**). However, CD27 does appear to have some utility in distinguishing BCG and TB treated individuals (AUC = 0.765), which increases slightly in combination with PD-1 (AUC = 0.814) (**Fig. 7C**). In conclusion, these models suggest that PD-1 and CD27 in combination are potential markers for distinguishing healthy individuals with latent Mtb infection (LTBI) from healthy individuals who have been previously treated for Mtb infection.

## Discussion

Latent infection with Mtb poses a threat to public health globally due to the presumed persistence of Mtb *in vivo*, which can lead to TB reactivation [3], [4], [5], [6]. CD4^+^ T cells have been shown to play a role in the control of Mtb infection [36]. In this study, we investigated how Mtb infection shapes the quality of the memory CD4^+^ T cells during latency by comparing the immune phenotype and function of long-lived antigen-specific memory CD4^+^ T cells in healthy BCG-vaccinated individuals who were either uninfected (BCG) or infected with Mtb (LTBI). We used Mtb cell wall (CW) antigens for stimulation of PBMCs from these two groups as the cell wall plays a critical role in Mtb intracellular survival, persistence, and pathogenicity, and provides a rich source of diverse antigens for immune recognition [37], [38].

LTBI and BCG individuals harbored resting, non-cycling effector memory populations that were not destined for apoptosis. This finding is in agreement with the idea that effector memory T cells in LTBI may reflect the continued presence of live, non-replicating bacteria [39]. Comparison of cytokine-expressing CD4^+^ T cells revealed significantly higher percentages for IFN-γ, TNF-α and IL-2 cells in LTBI as compared to BCG group. However, the long-lived memory CD4^+^ T cells in both groups had similar cytokine profiles that were predominantly polyfunctional with respect to IFN-γ, TNF-α and IL-2 production after CW stimulation. These results in LTBI were in agreement with recent studies in which similar proportions were detected using PPD, which is also a mixture of Mtb antigens [40], [41]. However, these results contrast with data using latency-associated antigens where predominantly monofunctional CD4^+^ T cells were reported [42], [43]. Studies of vaccine-induced immunity and chronic viral infections have suggested that polyfunctional T cells that produce multiple cytokines are more likely to provide protective immunity than T cells that produce a single cytokine [21]. While polyfunctional memory CD4^+^ T cells have been detected in healthy individuals with LTBI [40], [41], [44], [45], [46], [47], [48] and in BCG-vaccinated infants [49]; their role in providing protective immunity against TB remains unclear [19].

The major phenotypic difference between the LTBI and BCG groups was the expression of the costimulatory marker CD27 on antigen-specific memory CD4^+^ T cells. LTBI individuals harbored significantly higher frequencies of antigen-specific effector memory CD4^+^ T cells that had lost CD27 expression. Since loss of CD27 has been shown to be associated with antigen-induced maturation of effector memory subsets [31], these results suggest that the presence of Mtb in LTBI drives these subsets to later stage of differentiation than seen in the BCG group. This finding is in agreement with a previous study which showed that patients with smear and/or culture positive pulmonary TB had the highest percentages of CD27^−^ tuberculin-reactive CD4^+^ T cells followed by LTBI and then BCG-vaccinated individuals [50]. In addition, we show that whereas polyfunctional cytokine production in BCG individuals is mainly associated with CD27^+^, in LTBI both CD27^+^ and CD27^−^ subsets are polyfunctional, suggesting that CD27 does not reflect functional capacity. The memory CD4^+^ T cell phenotype in LTBI is distinct from latent cytomegalovirus (CMV) infection, where antigen-driven accumulation of terminally differentiated, CD27^−^ CD45RA^+^ effector memory CD4^+^ T cells have been described [51], [52]. Unlike the poorly proliferating CD27^−^ CD45RA^+^ T cells as well as CD27^−^ CD45RA^−^ T cells in latent CMV infection [53], [54], the CD27^−^ CD45RA^−^ subsets in LTBI display robust proliferative capacity *in vitro* in response to stimulation with CW antigens (T.A. and J.R., unpublished data). While latency-associated antigens have been shown to potentially be useful to discriminate between different stages of TB based on IFN-γ frequencies, the phenotypes of these antigen specific T cells in PBMCs or at the site of infection have not yet been elucidated [55], [56], [57], [58], [59].

PD-1 is a co-inhibitory receptor that is up-regulated on activated antigen-specific effector T cells in viral infections where it has been implicated in promoting dysfunctional responses [34], [35], [60]. PD-1 has also been shown to be expressed on Mtb-specific effector T cells in active TB patients [33]. In our study, the expression of PD-1 on antigen-specific memory CD4^+^ T cells is significantly higher in LTBI compared to the BCG group, suggesting that PD-1 expression may correlate with the presence of Mtb in latent TB. Our studies on PD-1 expression in latent Mtb infection show similarities to a recent report on latent viral infections [61]. This study showed that in healthy individuals with latent viral infections, PD-1 was expressed on functional EBV-and CMV- specific memory CD8^+^ T cells. In contrast, in cleared viral infections, PD-1 expression on influenza- and vaccinia-specific memory CD8^+^ T cells was significantly lower [61]. In LTBI, we found that antigen-specific PD-1^+^ T cells are polyfunctional suggesting that PD-1 expression does not affect the ability of CD4^+^ T cells to produce IFN-γ, TNF-α and IL-2. Similar results have been reported for SIV-specific CD8^+^ T cells [62]. Furthermore, the observed spread in the frequencies of PD-1^+^IFN-γ^+^ cells in LTBI is reminiscent of observations in non-human primate models where LTBI is represented by a spectrum of granulomatous lesions in the lungs of cynomolgus macaques [63]. Our study raises the interesting speculation that PD-1 levels on antigen-specific effector memory CD4^+^ T cells may reflect the proposed spectrum of latency in humans [6], [64], [65]. Interestingly, we found that PD-1 was preferentially expressed on cells that had lost CD27 expression and was therefore associated with the differentiation stage of CD4^+^ T cells. Moreover, there was a significant inverse correlation between the expression of PD-1 and CD27 across LTBI and BCG groups. Similar results have been reported in HIV infection where PD-1 expression was linked to the differentiation stage and activation status of HIV-specific CD8^+^ T cells [66].

Overall, our data suggest that the presence of CD27^−^PD-1^+^ antigen-specific effector memory CD4^+^ T cells may reflect persistent Mtb antigen stimulation *in vivo*. Alternatively, the heterogeneous expression of CD27 and PD-1 in LTBI and BCG groups may reflect the inherent phenotypic differences between the immune responses induced by Mtb and BCG at the time of infection or vaccination, which result in long-lived antigen-specific memory responses. This should be considered when developing new vaccines against TB. We also show, using ROC curve analysis and logistic regression models, that CD27 and PD-1 could predict LTBI and BCG status in healthy individuals. CD27 and PD-1 in combination also distinguish LTBI from healthy individuals who have been previously treated for TB and have successfully resolved Mtb infection. While our observations based on *in vitro* antigenic stimulation of PBMCs from LTBI, BCG and TB treated individuals do not necessarily reflect the *in vivo* situation within these individuals, they nonetheless provide a useful assay for discriminating between these sub-groups of healthy individuals. Our studies reinforce the advantages of T cell-based diagnostics like QuantiFERON and T-SPOT.TB over TSTs for identifying LTBI in BCG-vaccinated populations. Notably, while these assays eliminate issues of BCG cross-reactivity [4], [65], they do not distinguish LTBI individuals from healthy individuals who have been previously treated for TB. Incorporating CD27 and PD-1 as additional markers in T cell based assays thus has the potential to substantially improve our ability to evaluate true LTBI status. In the U.S., where 10–15 million individuals are estimated to have LTBI, targeting preventive treatment to the subset of individuals who are most likely to harbor Mtb *in vivo* is an important goal for TB control efforts.

## Materials and Methods

### Ethics Statement

This study was conducted according to the principles expressed in the Declaration of Helsinki. Ethical approval was obtained from the Emory University Institutional Review Board and all participants were provided written informed consent for the collection of samples and subsequent analyses.

### Study participants and PBMC isolation

Eighty healthy asymptomatic individuals (23 to 55 years of age) who have BCG vaccination histories were recruited at Emory University, Atlanta, GA. These individuals had no history of active tuberculosis, no recent history of severe respiratory diseases, a normal chest X-ray, and were anti-tuberculosis treatment-naïve and HIV negative. Twelve individuals who were previously treated for pulmonary active TB were recruited from Grady Hospital in Atlanta, GA. These individuals were originally diagnosed as to have active pulmonary TB by positive chest X-ray, positive sputum smear and/or culture and/or nucleic acid amplification tests. At the time of enrollment in the study, all TB-treated individuals were HIV negative, sputum-negative and had normal chest X-ray and were enrolled ≥2 years after completion of anti-tuberculosis treatment. Their status was confirmed by assaying for the presence of IFN-γ^+^ CD4^+^ T cells by intracellular cytokine staining in PBMCs stimulated with Mtb CW antigen and ESAT-6/CFP-10 peptide pools and compared with the profile of non-stimulated cells. Five non-BCG-vaccinated healthy individuals with no history of Mtb exposure were also recruited as controls. PBMC were isolated from blood using cell preparation tubes (CPT, BD Biosciences) and cryopreserved in 90% fetal FBS (Hyclone, South Logan, UT) and 10% dimethyl sulfoxide (Sigma-Aldrich, St. Louis, MO).

### IFN-γ ELISPOT assays

All antibodies for ELISPOT assays were purchased from Mabtech (Cincinnati, OH) and performed according to standard procedures. PBMC were plated in triplicate at 2×10^5^ cells/well and stimulated for 24 h at 37°C with 5 µg/ml Mtb cell wall (CW) antigens (NIH-TBVRM contract, Colorado State University), 5 µg/ml ESAT-6 and CFP-10 peptide pools synthesized by Genemed Synthesis Inc., (San Antonio, TX), as previously described [67]; 1 µg/ml SEB as the positive control or medium as the negative control. Spot-forming cells (SFCs) were counted on an automated ELISPOT reader (CTL series 3A analyzer, Cellular Technology, Cleveland, OH) and analyzed with Immunospot (CTL Analyzers). Responses were scored as positive if the test wells contained a mean of at least 10 SFCs more than the mean of the negative control wells.

### Cell stimulation, staining and flow cytometry

Before stimulation, cryopreserved PBMC were thawed and resuspended overnight at 37°C, 5% CO_2_ in RPMI-1640 medium (Lonza, Walkersville, MD) containing 10% FBS, 2 mM glutamine, 100 IU/ml penicillin, and 100 µg/ml streptomycin. The viability of the lymphocytes was 80–95%. 1×10^6^ PBMC were each stimulated with CW antigens (10 µg/ml) and ESAT-6 and CFP-10 peptides (10 µg/ml) at 37°C, 5% CO_2_ for 2 h followed by the addition of Brefeldin A (10 µg/ml) (BD Biosciences, San Diego, CA) and further incubated for 16 h. PBMC were washed, surface-stained with appropriate antibodies, permeabilized with Cytofix/Cytoperm Kit (BD Biosciences), stained intracellularly with appropriate antibodies: IFN-γ (clone B27), TNF-α (clone Mab11), IL-2 (clone MQ1-17H12), Ki-67 (clone B56), Bcl-2 (clone Bcl-2/100) and fixed with 1% paraformaldehyde before acquisition on a FACSCalibur (BD BioSciences) or LSR-II system (BD Biosciences). Monoclonal antibodies were obtained from BD Biosciences, eBiosciences (San Diego, CA), Beckman-Coulter (Fullerton, CA), R&D Systems (Minneapolis, MN) and Biolegend (San Diego, CA). FACS data were analyzed with FlowJo software (Tree Star Inc., San Carlos, CA). For multifunctional cytokine analysis, CD4^+^ T cells were gated on cytokine-positive cells. Boolean combination gating was performed to calculate the frequencies corresponding to seven different combinations of cytokines.

### Statistical analysis

Statistical analyses were performed using Graphpad Prism 4.0 software; the Mann-Whitney U test and the unpaired t-test were used. A p-value of less than 0.05 was considered to be statistically significant. The Pearson correlation test was used for correlation analysis. For the predictive models, data analysis was performed in the statistical software R [68]. For single marker analysis, Receiver Operating Characteristic (ROC) curve analysis was used which plots sensitivity (true positive rates) versus 1-specificity (false positive rates) for each marker individually. The area under curve (AUC) was determined by numerical integration for each marker [69] to evaluate the predictive value of the marker. An AUC value of 1 indicates the maximum predictive value for a given marker. To combine both markers for prediction, logistic regression was performed and the associated ROC was plotted, where the sensitivity and specificity were found as a function of the probability cutoff.

## Supporting Information

Figure S1 **CD4^+^ T cells from healthy controls (non-BCG-vaccinated donors) do not show IFN-γ^+^ reactivity to Mtb CW antigens.** PBMC from healthy controls or LTBI donors were either not stimulated (NS) or stimulated with CW or SEB and stained for flow cytometric analysis as described in the Methods.
(TIF)

Figure S2 **PD-1 levels on unstimulated PBMC do not differ between LTBI, BCG and TB treated groups.** PBMC from each of the three groups LTBI, BCG, TB-treated) were stained with PD-1 and analyzed by flow cytometry. No significant differences in the frequencies of PD-1-expressing CD4^+^ T cells were observed.
(TIF)
