# Supplementary figures and images for: Distinct Effector Memory CD4+ T Cell Signatures in Latent Mycobacterium tuberculosis Infection, BCG Vaccination and Clinically Resolved Tuberculosis
Source: PLoS One. 2012 Apr 24;7(4):e36046. doi: 10.1371/journal.pone.0036046 (PMC3335801; doi:10.1371/journal.pone.0036046)

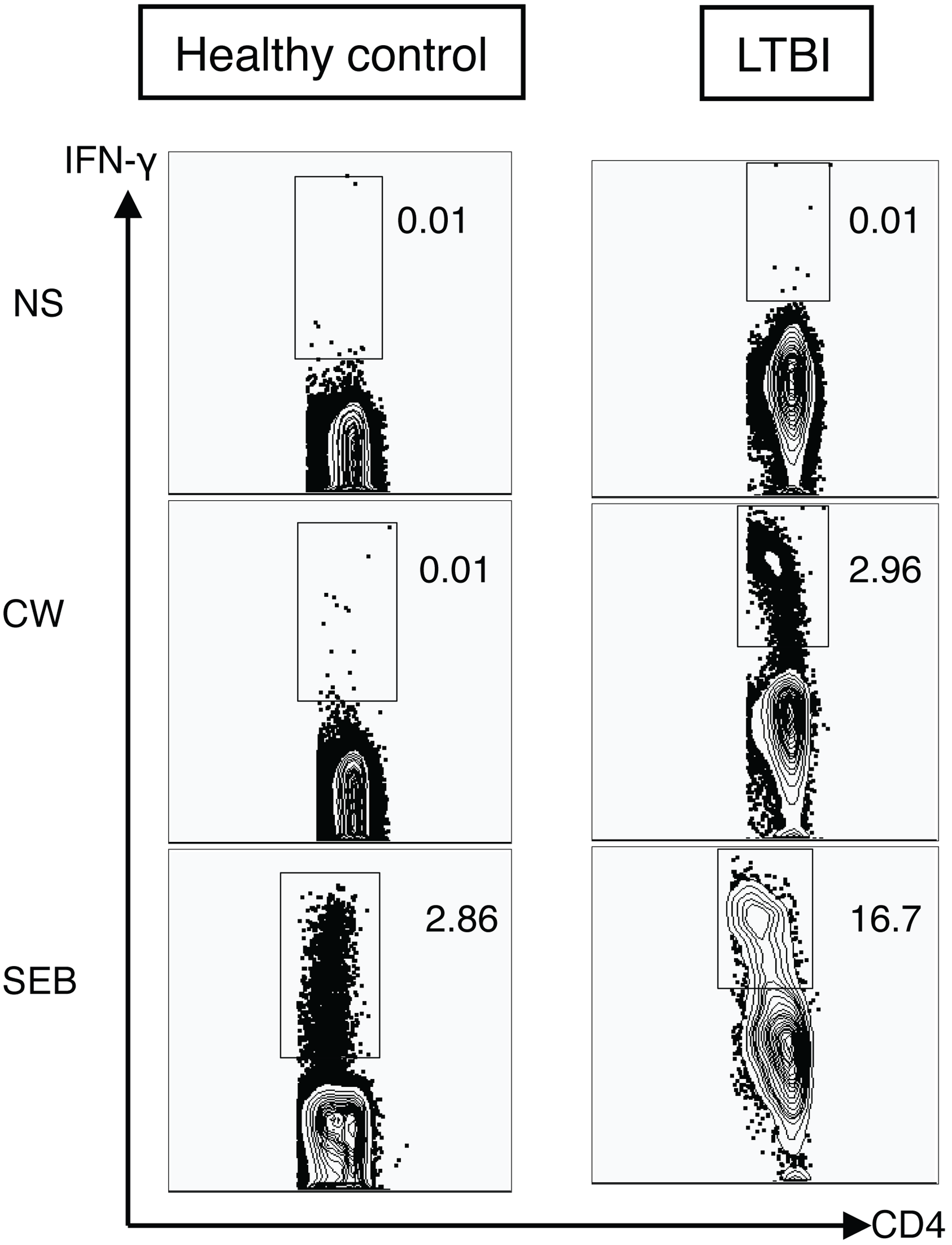

Supplement: Figure S1 — CD4+ T cells from healthy controls (non-BCG-vaccinated donors) do not show IFN-γ+ reactivity to Mtb CW antigens. PBMC from healthy controls or LTBI donors were either not stimulated (NS) or stimulated with CW or SEB and stained for flow cytometric analysis as described in the Methods. (TIF) [file pone.0036046.s001.tif]

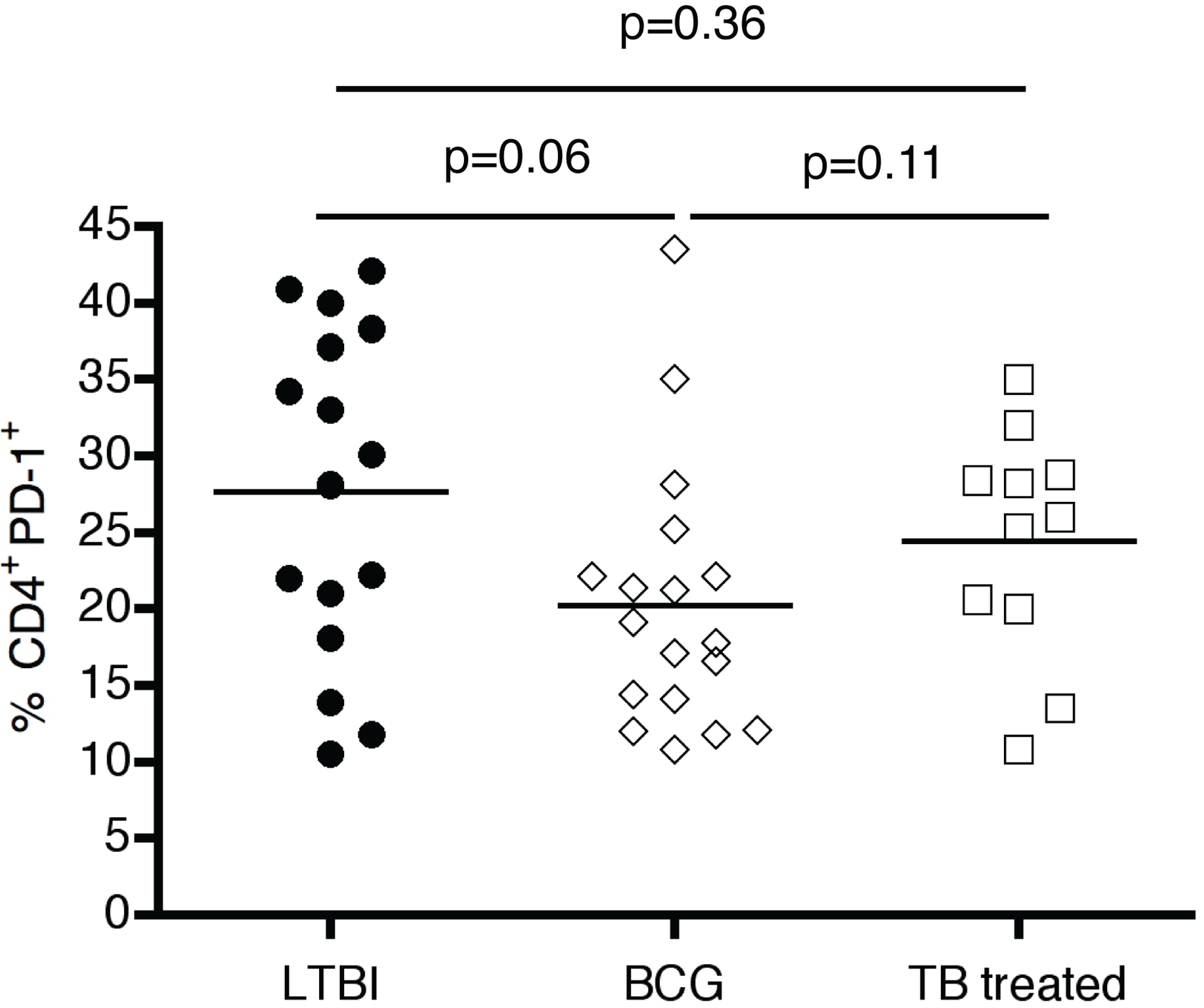

Supplement: Figure S2 — PD-1 levels on unstimulated PBMC do not differ between LTBI, BCG and TB treated groups. PBMC from each of the three groups LTBI, BCG, TB-treated) were stained with PD-1 and analyzed by flow cytometry. No significant differences in the frequencies of PD-1-expressing CD4+ T cells were observed. (TIF) [file pone.0036046.s002.tif]
